# Calibration of Drucker–Prager Cap Constitutive Model for Ceramic Powder Compaction through Inverse Analysis

**DOI:** 10.3390/ma14144044

**Published:** 2021-07-20

**Authors:** Vladimir Buljak, Severine Baivier-Romero, Achraf Kallel

**Affiliations:** 1 Strength of Materials Department, Mechanical Engineering Faculty, University of Belgrade, 11000 Belgrade, Serbia; 2 Global Fundamental Research Manager—Vesuvius, 7011 Ghlin, Belgium; Severine.Romero.Baivier@vesuvius.com (S.B.-R.); Achraf.Kallel@vesuvius.com (A.K.)

**Keywords:** powder compaction, Drucker–Prager Cap model, inverse analysis, refractory, material characterization, yield surface

## Abstract

Phenomenological plasticity models that relate relative density to plastic strain are frequently used to simulate ceramic powder compaction. With respect to the form implemented in finite element codes, they need to be modified in order to define governing parameters as functions of relative densities. Such a modification increases the number of constitutive parameters and makes their calibration a demanding task that involves a large number of experiments. The novel calibration procedure investigated in this paper is based on inverse analysis methodology, centered on the minimization of a discrepancy function that quantifies the difference between experimentally measured and numerically computed quantities. In order to capture the influence of sought parameters on measured quantities, three different geometries of die and punches are proposed, resulting from a sensitivity analysis performed using numerical simulations of the test. The formulated calibration protocol requires only data that can be collected during the compaction test and, thus, involves a relatively smaller number of experiments. The developed procedure is tested on an alumina powder mixture, used for refractory products, by making a reference to the modified Drucker–Prager Cap model. The assessed parameters are compared to reference values, obtained through more laborious destructive tests performed on green bodies, and are further used to simulate the compaction test with arbitrary geometries. Both comparisons evidenced excellent agreement.

## 1. Introduction

The ceramic industry includes the production of various components with different engineering applications, ranging from simple ones, such as tiles and bricks, up to more sophisticated ones, represented by refractory products, aerospace components, devices for bio-applications, etc. Considering the evident industrial interest, a significant research effort has recently been devoted to the development of models to be used for reliable simulations of different production phases (e.g., see [1,2,3,4,5,6,7,8,9]). Such simulations are of major importance in the manufacturing of advanced components, such as those used in refractory applications for the control of liquid iron flow, those designed to work in aggressive environments and those subjected to severe thermo-mechanical loadings [10,11].

Ceramic parts are commonly produced by a two-step process [12]. Within the first step, the component is shaped through the compaction of powder material in order to form the so-called “green body”, which is then, in the second step, subjected to sintering at a high temperature. This manufacturing path may induce a density gradient in the final part, or more severe internal voids or defects. A lack of density homogeneity within the green body directly influences the local shrinking during the sintering phase, while internal defects may lead to crack initiation and subsequent damage of the structural component. Obtaining high homogeneity is therefore required, and is achievable through the optimization of tool geometry. In order to avoid trial and error approaches when performing this optimization, it is desirable to have a reliable numerical model of the compaction process.

An important ingredient of this numerical modeling represents the constitutive description of a powder involved within the process. Such constitutive modeling is rather challenging, as the material changes its mechanical response from a powder loose state, to a dense one, typical of a solid body. Several authors have developed micro-mechanical models in order to study the evolution of the contact pressure between grains at the particle scale (see e.g., [4,5,6]). In order to capture the effects of particle rearrangement, deformation and pore filling, the modeling is based on a discrete element method ([5,6]) or a multi-particle finite element method [7,8]. These models have to be multi-scale, in view of their application for large component simulations, with particle-scale simulations used to generate constitutive equations for large scale continuum-based models [13]. Such a circumstance makes both the numerical implementation and calibration laborious tasks, where the latter frequently requires a large number of experiments [6].

Alternatively, continuum-based models using the traditional finite element method can be used for the simulations, offering the advantage of solving the problem on a single scale. These models proved to be effective in predicting local density and stress distributions within the green body and are often adopted as a more robust alternative to multi-scale approaches [13,14,15,16]. Using the assumption that within the compacted green body at high relative densities the discontinuity of powder becomes irrelevant [14], its mechanical behavior can be described by phenomenological models used in soil mechanics. Following this idea, there have been diverse applications employing models such as the Mohr–Coulomb [15], Cam-Clay [17] and Drucker–Prager Cap model [15], or, when elasto-plastic coupling becomes important, a modified Drucker–Prager Cap model [13,14,18]. The Drucker–Prager Cap model represents one of the models with the largest flexibility, as its application is used, aside from powder, for the modeling compaction process of broken wooden waste materials [19].

With regards to the reliability of the computational results, regardless of the numerical strategy used to model the powder compaction, the accuracy of the inputs in terms of governing parameters is crucial. As the current praxis, destructive experiments are performed on green bodies for calibration purposes. In [14], Han et al. used Brazilian disk tests together with an uniaxial compression test to assess shear failure surface within the modified Drucker–Prager Cap model. A similar approach is adopted by Sinha et al. [15] within the study of the sensitivity of Drucker–Prager Cap parameters during the unloading phase. Such approaches are more laborious when employed to capture the evolution of parameters over a range of relative densities, requiring the testing of an elevated number of green bodies with different relative densities. Addressing the problem of the identification of parameters as a function of relative density, Garner et al. [20] proposed the scheme for the parameter extrapolation of relative density levels that are difficult to test. In a more recent study, Khoei [18] showed that by employing radial stress measurements collected during the compaction tests, cap surface parameters can also be calibrated. The assessed parameters are further verified through the numerical models used to simulate the compaction test.

Melo et al. [21] used a more sophisticated approach in parameter identification based on inverse analysis by considering data from a measured pressure. The best parameter set was selected as the one that minimizes the discrepancy between measured and simulated data. To keep the number of parameters to be identified relatively small, a simpler version of the Drucker–Prager Cap model was used without elasto-plastic coupling and with simpler hardening that only affected the cap surface. Zhou et al. [22] also used an inverse analysis-based approach, where only the pressing curve was used as the experimental data. In their study, a simpler constitutive model without elasto-plastic coupling was also consid77ered. Atrain et al. [23] also made reference to the Drucker–Prager Cap model, also introducing elasto-plastic coupling. They calibrated the model on the bases of artificial neural networks. Penasa et al. [24], on the other hand, employed a rather complex model for the powder compaction with a yield surface that changes its shape with the compaction. They evidenced that, when the model with such a high complexity is employed, its calibration becomes a demanding task. For the simulation of simple experiments (e.g., a cylinder under uniaxial strain compaction) it was shown that multiple material parameter sets could fit the same experiment.

In most of the previously mentioned earlier studies, destructive experiments on green bodies were required in order to provide constitutive parameters. The use of the numerical simulation of the compaction process was restricted to merely verification purposes, without its systematic incorporation within the calibration procedure. It is therefore clear that the calibration of such models still represents a practically meaningful challenge that arises in diverse areas of the application of powder compaction. Newer studies that employ an inverse analysis for the parameter calibration evidenced certain numerical challenges that the procedure has to face. Hence, simplified constitutive models are commonly used (such as in [21,22]). From the point of view of the inverse analysis, if more complex models with a larger number of parameters are being employed (such as in [24]), the problem becomes ill-posed when only simple experiments are considered (e.g., the uniaxial pressing of a flat cylinder).

The contribution presented herein to the above outlined problem consists of designing a procedure aimed at identifying constitutive parameters by using only measurable quantities that can be collected from compaction tests. These experimental data are employed as the input of an inverse analysis procedure, centered on a minimization of a discrepancy function, which quantifies the difference between measured and computer quantities. Thus, constitutive parameters of complex models can be assessed only by performing compaction tests, avoiding any further experimentation on green bodies. In contrast to certain previous studies that used a similar approach (e.g., [21,23,24]), a larger diversity of experimental data is included in this study by simultaneously considering different geometries within the calibration phase. This diversity should serve to “activate” all the material parameters so that their assessed values can be treated as a representative one, rather than fitting a single experiment only. In order to ascertain the influence of sought parameters to the measurable quantities (here, specifically the force–displacement relationship), numerical sensitivity analyses are first performed. Such analyses result in the formulation of three different die/punch geometries, which prove to be sufficient to calibrate the considered constitutive model.

Within the novel procedure that is proposed and investigated in what follows, a reference is made to the modified Drucker–Prager Cap (DPC) model, though the methodology presented can be applied also to other constitutive models used for powder compaction simulations. With respect to some previous studies ([13,14,19,20,21,22,23]), the modification of the model proposed here includes elasto-plastic coupling and a more flexible definition of hardening that also affects the shear failure surface. For validation purposes, the assessed values of selected parameters are compared against those obtained through traditional destructive tests performed on the green bodies. Furthermore, parameters obtained through the inverse analysis procedure are used as inputs for “direct” analysis (i.e., numerical simulation of the compaction test), with complex geometries in order to generate force vs. displacement curves, which are subsequently compared to the experimental ones. A similar comparison was also included in [23]. The verification geometry used there was a hollow cylinder while the one used for calibration was a flat cylinder. Such a comparison may result in a pre-conditionally good match, due to a fairly similar stress state distribution over the sample. To test the procedure in a more demanding stress distribution, here, three different shapes are considered for comparative purposes. The comparative analysis serves as a quantitative basis for the discussion of advantages and limitations of the proposed method.

## 2. Modified Drucker–Prager Cap Constitutive Model and Relevant Parameters

The material model adopted in the present study is a modified DPC model, as proposed by several authors [13,14,15]. In addition to the version implemented in commercial software ABAQUS [25], a modification of the model is employed by defining the governing parameters as a function of volumetric plastic strain, which, in turn, can be related to the relative density of a specimen. The modification is introduced through a user subroutine (USDFLD) available in ABAQUS, to formulate solution-dependent parameters.

**Figure 1:**
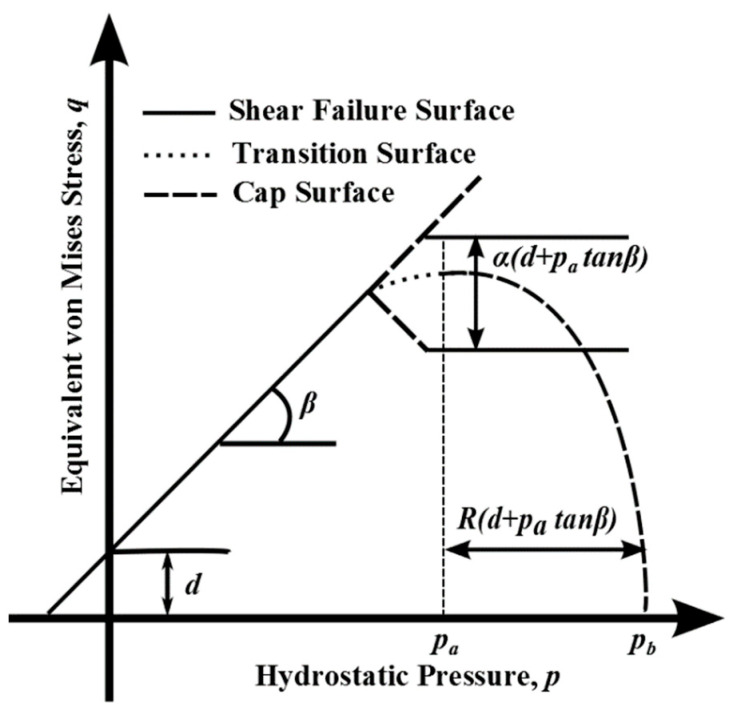
Drucker–Prager Cap yield surface in meridian plane.

The DPC yield surface is enclosed by three surfaces: a shear conical surface (present in classic form of DP yield criterion), an elliptical surface (called “cap”) and a transition surface between the two, for establishing a smooth transition in order to avoid numerical instabilities. The yield condition consists of three separate equations$:f_{s}\left(\sigma \right)$, $f_{C}\left(\sigma \right)$ and $f_{T}\left(\sigma \right)$ (letters in subscript refer to shear surface, cap surface and transition surface, respectively):

(1) $$f_{s}\left(\sigma \right)=q-\tan\left(\beta \right)p-d=0$$

(2) $$f_{C}\left(\sigma \right)=\sqrt{{\left(p-p_{a}\right)}^{2}+{\left[\frac{R\cdot q}{1+\alpha -\alpha /cos\beta }\right]}^{2}}-R\left(d+p_{a}tan\beta \right)=0$$

(3) $$f_{T}\left(\sigma \right)=\sqrt{{\left(p-p_{a}\right)}^{2}+{\left[q-\left(1-\frac{\alpha }{cos\beta }\right)\left(d+p_{a}tan\beta \right)\right]}^{2}}-R\left(d+p_{a}tan\beta \right)=0$$

where *p* and *q* are hydrostatic pressure and the equivalent von Mises stress, respectively. Assuming the associate plastic flow rule on cap, and non-associative on the shear surface and transition surface, the DPC model is completely defined by quantifying five constitutive parameters used within the yield condition given by (1)–(3): the material cohesion *d* and friction angle *β* required to define shear surface, the “cap eccentricity” *R* and the evolution pressure *p_a_* (or alternatively, the hydrostatic pressure yield stress *p_b_*, see Figure 1) to define the shape and position of the elliptical cap and parameter *α* that governs the transition region (i.e., the connection between the cap and shear failure surface).

Particularization of the above outlined model adopted in this study is related to the implementation of parameter evolution within the compaction process. In a typical theory of plasticity jargon, the change in yield criterion governing parameters with accumulated plastic strain is referred to as “hardening”. In the present study, a specific form of hardening is introduced, where parameters are assumed to depend on relative density. Volumetric plastic strain, taken as an internal variable on which governing parameters depend, is related to the relative density through the following equation:

(4) $$\varepsilon_{p}^{V}=ln\left(\frac{\rho }{\rho_{0}}\right)$$

where *ρ* and *ρ*_0_ represent the current and initial (i.e., corresponding to zero plastic strain) relative density. Unlike the other studies [14,15,16,20,24] that used some form of modified DPC model, more extended modification is proposed here. The adopted relationship between parameters and relative density is proposed by the following exponential dependency:

(5) $$P\left(\rho \right)=\left(P_{1}-P_{0}\right){\left(\frac{\rho -\rho_{0}}{\rho_{1}-\rho_{0}}\right)}^{n}+P_{0}$$

where *ρ*_0_ and *ρ*_1_ are fixed values of relative density (specifically 0.5 and 0.95 respectively) while *P*_0_ and *P*_1_ are corresponding values of a particular parameter at these relative densities, and *n* is the exponent. Through Equation (5), the dependency of four plastic parameters (namely *d*, *β*, *R*, *p_b_*) on relative density is introduced, while for the transition radius (α), a constant value of 0.025 is assumed, as this parameter proved to have a marginal effect on simulated test output, and is introduced for numerical stabilization purposes. The selection of this value is motivated by the suggestion given in ABAQUS User’s Manual [25] regarding its range: specifically, between 0.01 and 0.05. The same dependency on relative density is also assumed for the elastic parameters, Young’s modulus (*E*) and Poisson’s ratio (*ν*).

The above outlined definition of constitutive parameters as a function of solution-dependent field variable formulates the modified DPC model. The model takes into account the elasto-plastic coupling, while implemented hardening on plastic parameters is general enough to account for both linear and exponential relations, governed by the parameter *n*, subjected to the identification together with other parameters. Unlike the most common form of the DPC model, here, the adopted modification defines hardening, which also affects the shear failure surface, which becomes important for certain powders (see [24]).

The identification procedure proposed here consists of the assessment of three values for each of four plastic and two elastic material parameters: its value at low density, at high density and the exponent of transition (quantities *P*_0_, *P*_1_ and *n* in the Equation (5)). The coefficient of the Coulomb friction between the die wall and specimen is also subjected to the identification. In most previous studies, its value was assumed as a priori known (see e.g., [14,20]). It is evidenced in the literature that friction strongly affects the force–displacement curve measured during the compaction test (see [26] and references therein). Within the proposed method, it is possible to include the friction coefficient among the set of sought parameters, resulting in more accurate material parameter quantifications, with no special care required in the experiment to reduce the influence of friction (such as the one proposed in [14]).

## 3. Material Parameter Quantification Based on Inverse Analysis

The identification procedure proposed here is centered on the minimization of the suitable discrepancy function, which quantifies the difference between experimentally measured quantities and their numerically computed counterparts. In a purely deterministic context, this function is formulated as:

(6) $$\omega \left(p\right)={\left[u_{e}-u\left(p\right)\right]}^{T}\left[u_{e}-u\left(p\right)\right]$$

with experimentally measured quantities collected in vector ***u****_e_*, and corresponding computed values forming the vector ***u***(***p***), that, through test simulations, are related to the vector of parameters ***p***. The selection of measurable quantities is motivated by sensitivity analyses, described in the following section. The resulting minimization problem is solved on the basis of an iterative mathematical programming algorithm relying on a trust region (TR) strategy. Such an algorithm is fairly effective when the function to be minimized is convex (i.e., is not featured by large number of local minima), which proves to be the case in the present context. The main features of the TR applied here are briefly outlined in what follows, while the reader should refer to [27,28] for details.

The iterative sequence starts by attributing to the parameters certain “guess” values. In this initialization point (***p****_k_*), first, derivatives are computed through finite differences (i.e., by separately perturbing each of the parameters). The model function is generated as a quadratic approximation of the discrepancy function, based on a computed gradient, and second derivatives are approximated through a Jacobean, namely:

(7) $$\omega_{k}\left(p_{k}\right)=\omega \left(p_{k}\right)+p_{k}^{T}\;\nabla \omega \left(p_{k}\right)+\frac{1}{2}p_{k}^{T}\nabla^{2}\omega \left(p_{k}\right)p_{k},\;\;\mathrm{where}\;\nabla^{2}\omega \left(p_{k}\right)=J^{T}J$$

The selected optimization algorithm relies on the calculation of first derivatives and the approximation of second derivatives, which tend to be fairly accurate within the vicinity of the solution (see [28]). It provides an almost quadratic convergence and generally outperforms direct search methods, such as the Nelder–Mead method applied in [22].

The minimization of the model function within the bounds of the trust region leads to a new parameter vector corresponding to the reduced value of the discrepancy function. In the new point in the parameter space, the model function is again constructed, where the process iteratively repeats upon reaching the prescribed convergence criteria. For the effective implementation of this identification procedure, two aspects are crucial: the sensitivity of measurable quantities to sought parameters, and the verification of uniqueness of the obtained solution.

### 3.1. Experiment Configuration and Selection of Measurable Quantities

**Figure 2:**
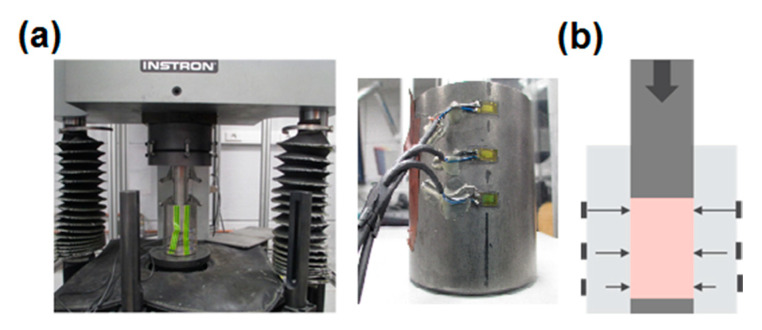
(**a**) Pressing machine used for formation of green bodies, location of radial stress measurements; (**b**) schematic representation of cylindrical greed body with pressure sensor locations.

**Figure 3:**
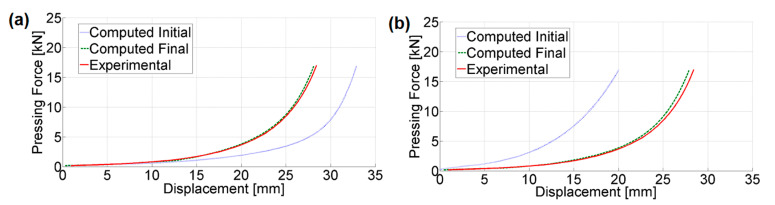
Computed test response vs. experimental response from two different inverse analysis results: (**a**) initialization 1; (**b**) initialization 2.

The experiment is performed on a pressing machine, visualized in Figure 2, while the geometry of the formed green body can be an arbitrary one. A typical output of the compaction experiment is a force vs. displacement curve, with the form as shown in Figure 3. The simplest possible shape to consider is cylindrical, schematically presented in Figure 2b.

The inverse problem constructed by considering the force vs. displacement curve taken from the compaction of a cylindrical sample as experimental data proves to be ill-posed, where the discrepancy function does not have only one well-defined global minimum. Therefore, the iterative TR procedure converges to fairly different parameter sets when starting from diverse initialization points, with all converged sets fitting the experimental curve well. Figure 3 shows two representative examples, comparing the computed response with the experimental one, which corresponds to the parameter sets obtained by solving the inverse problem with two different initializations. Both of these parameter sets are valid solutions to the inverse problem, since they both minimize the discrepancy function to the same extent. Neither of the two parameter sets are representative material parameter sets, due to the considerable difference in parameter values. In Table 1, the selected parameter values from the two sets are shown, corresponding to the parameters with the largest difference.

Previous considerations corroborate the conclusion that the selected experiment is too simple to be used for calibration of the considered model, mainly caused by the fact that the sample shape produces an almost uniform stress distribution within the sample. It is therefore desirable to define the shapes of the tested green bodies in order to provoke a more heterogeneous stress distribution. The selection of the shape is motivated on the basis of the results of the sensitivity analysis performed through test simulations.

### 3.2. Sensitivity Analysis and Proposed Test Configurations

Given the large complexity of the considered constitutive model, the regularization of the inverse problem is achieved through the simultaneous use of multiple experimental configurations. Aside from the simple cylindrical shape, hereafter referred to as configuration 1, additional configurations are proposed.

**Figure 4:**
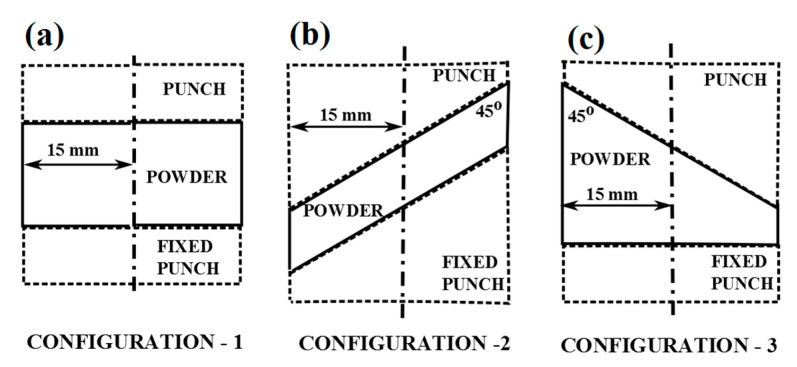
Adopted configurations for calibration through powder compaction tests: a flat cylinder (**a**), an inclined cylinder (**b**) and a combination of the two (**c**).

The major difficulty related to the sole use of configuration 1 is that the shear component of the stress in the whole specimen is negligibly small. Hence, the specimen response to this test is not influenced by shear failure surface parameters. To circumvent this shortcoming, a configuration presented in Figure 4b (hereafter referred to as configuration 2) is selected, where the zone is within the specimen with a dominant shear component of stress.

The quantification of the sensitivity of measurable quantities from the test, with respect to shear failure surface parameters, is achieved by performing a sensitivity analysis in its traditional meaning (see e.g., [29,30]). Specifically, it is provided through the computation of a partial derivative of a specific measurable quantity, with respect to a sought parameter. Therefore, the sensitivity of measurable quantity *q_i_*, with respect to the parameter *p_j_*, expressed as a normalized and dimensionless value, is computed through:

(8) $$S_{ij}=\frac{\partial q_{i}}{\partial p_{j}}\cdot \frac{p_{j}}{q_{j}}$$

**Figure 5:**
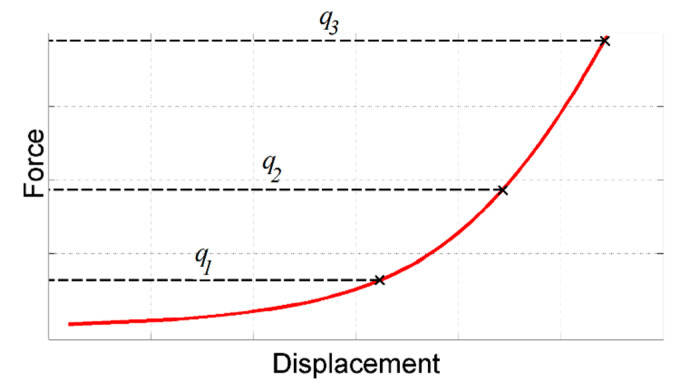
Measurable quantities selected for sensitivity analysis.

Comparative results confirm a significantly larger sensitivity, with respect to the shear failure surface parameters, of measurable quantities collected from the test with configuration 2 than those from configuration 1. Table 2 visualizes results regarding cohesion at a relative density of 0.95. Results consider three measurable quantities: displacement values corresponding to 20%, 50% and 100% of the maximum test force within the test (see Figure 5). The derivatives are computed through finite forward differences, perturbing the parameters by 10%. Similar results are obtained for the friction angle (*β*), exhibiting an approximately one order of magnitude larger sensitivity related to measurable quantities collected from configuration 2.

**Figure 6:**
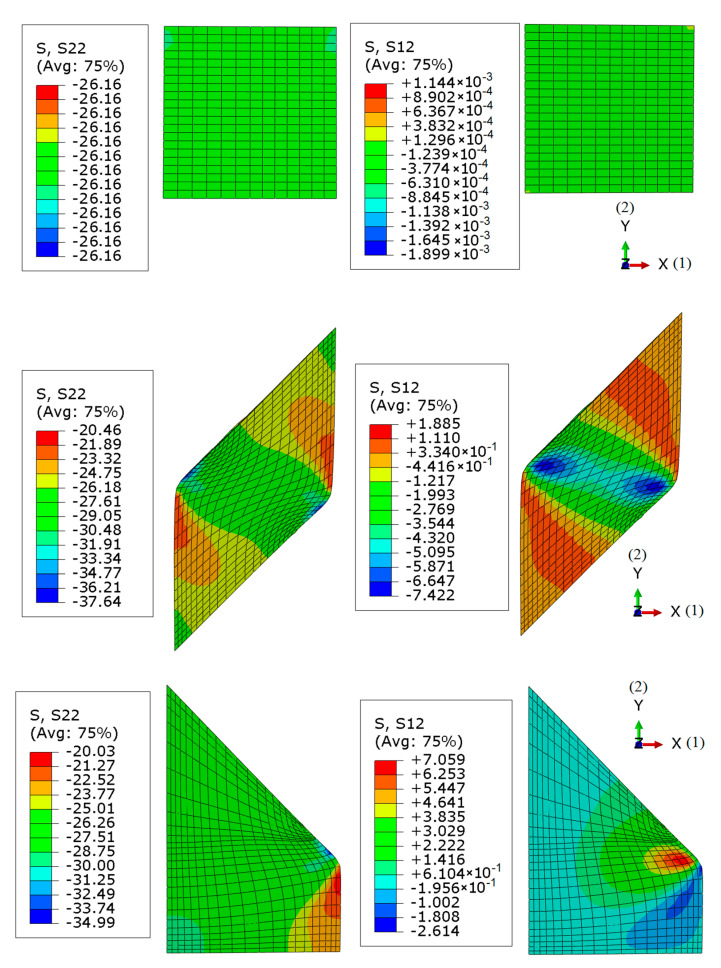
Stress distribution for axial and shearing stress for selected configuraions.

**Figure 7:**
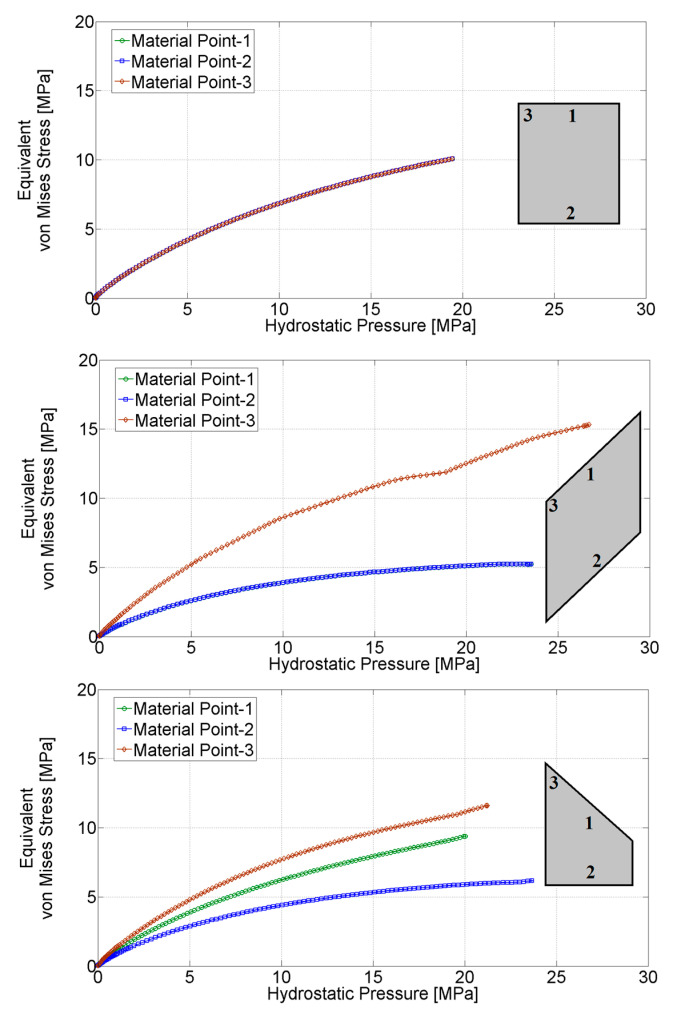
Stress path for selected material points.

In order to ensure the influence of all sought parameters on the measurable quantities from the test, it is important to make the stress field over the specimen strongly non-uniform. For this purpose, configuration 3, illustrated in Figure 4c is adopted. Numerical simulations of the tests employing three configurations visualized in Figure 4 are performed in order to verify the achieved result. Figure 6 shows the stress distribution over the samples corresponding to three selected configurations at the end of the compaction process. The distributions of the stress components σ_22_ and σ_12_ are visualized for all three configurations. The different material points of the stress “paths” that are passing through the compaction process are compared for all three configurations. The results are summarized in Figure 7, showing the stress path for three material points (with their location pointed in the figure). From these figures, it can be seen that the stress distribution is significantly more non-uniform in configurations 2 and 3 than in configuration 1, with the largest diversity of stress paths between considered material points obtained in configuration 3.

Numerical simulations, which led to the results given in Figure 6 and Figure 7, are performed in a frictionless regime in order to comparatively investigate the influence of the specimen geometry only. It is evidenced in the literature that the effect of friction between the powder and die wall influences the stress distribution, and, consequently, the measured force–displacement curve [14,31]. The assessment of the correct value of the friction coefficient is therefore rather important. In the present study, it is subjected to the identification together with other parameters. To capture the information about the vertical stress gradient (i.e., along the compaction axis), radial stresses are measured in three points along the height of the die at its outer surface (see Figure 2).

The results of the above investigations led to the formulation of a calibration protocol, briefly outlined as follows:

1. Three compaction tests are performed by employing test configurations shown in Figure 4. From all three tests, force vs. displacement curves are collected together with radial stresses measured at three different points at the outer surface of the mold (see Figure 2);
2. Computer simulations of the three tests are carried out in order to generate the computed counterpart of the above measured quantities. From each test, 100 displacements corresponding to 100 equally spaced force levels starting from zero up to the maximum value used in the test are collected, with 100 radial stress values for each of the three measuring points, corresponding to the same 100 force levels. An account is taken of the normalization for the diverse orders of magnitude of different entries;
3. A discrepancy function is constructed in the form given by (6), using the experimentally measured and numerically computed quantities, collected in vectors ***u****_e_* and ***u***(***p***), respectively.
4. The formulated minimization problem is solved by employing the iterative TR algorithm described earlier. In view of the possible local non-convexity of the discrepancy function, resulting in convergence to a local minimum, the reliability of the solution achieved after the TR convergence can be verified by performing the minimization with diverse initializations.

The proposed calibration protocol represents a good framework to assess representative material parameters on the basis of experimentally measured data. Representative here means that the material properties assessed through the proposed identification procedure can give a reliable numerical simulation of an arbitrary geometry of the green body, which is verified and presented in Section 5. Data required as inputs for the outlined procedure are collected from the experiments performed on alumina powder. Details of the employed experiments and used materials are given in the following section.

## 4. Materials and Experiments

**Figure 8:**
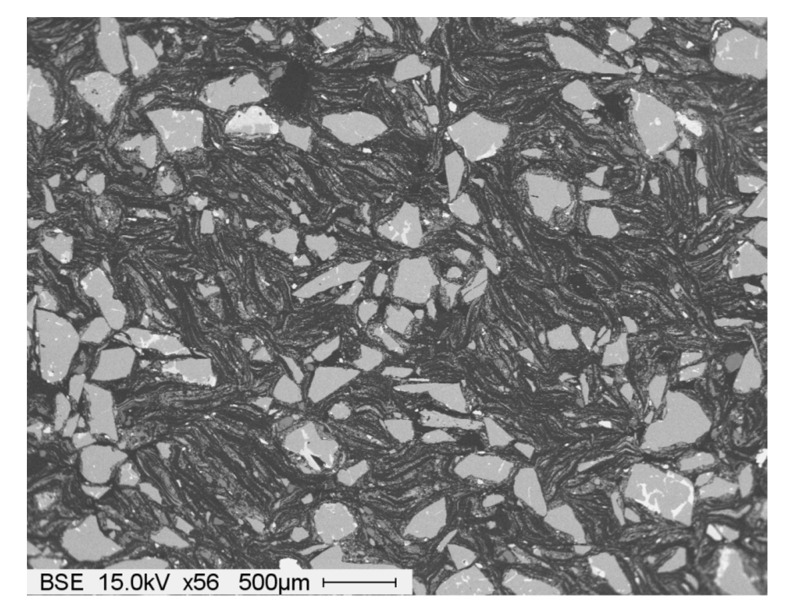
Microstructure of pressed powder.

The studied material is a mixture of hard alumina particles and graphite leaf embedded into a soft carbonic matrix. The alumina and graphite volumetric ratios are equal to 40% each, with 20% of binder used. At the end of the mixing process, millimetric composite pellets are obtained. The microstructure of pressed powder is visualized in Figure 8, evidencing a homogeneous repartition of the mixture compounds.

The powder was loaded into a stainless steel die and then compacted uniaxially with a constant velocity of 1.5 mm/s. For studied powder, no strain-rate effects were noticed for this range of compaction velocity. The instrumented tooling used in this work is presented in Figure 2. The device deformation during calibration test was measured up to 200 MPa and eliminated from the results in order to extract just the intrinsic behavior of tested powder. This was achieved by employing the procedure described in [32]. No special care was taken within the experiment to reduce the friction, with friction coefficient also subjected to the identification.

To measure radial stresses, three strain gages were glued to the die at different levels (see Figure 2). Measured radial deformation is related to the pressure the sample transferred to the internal radial die surface. “Calibration” of the measurement was carried out on the basis of pressing experiments on rubber samples with different heights, assuming to have Poisson’s ratio of almost ν = 0.5, and therefore being incompressible. The calibration was performed up to the axial stress value of 200 MPa.

**Figure 9:**
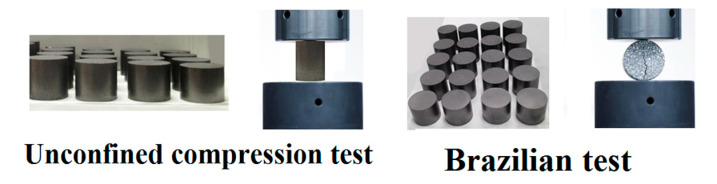
Destructive tests performed on green bodies.

In order to establish reference values for selected parameters for comparative purposes, where this procedure is described in [14], the reliance on destructive tests of green bodies was employed. A series of cylindrical samples with different values of relative density were prepared. Samples with height to diameter ratio of 2:1 were tested with unconfined compression test (i.e., crash test), while samples with height to diameter ratio of 1:2 were subjected to Brazilian test (see Figure 9). On the basis of these tests, parameters defining shear failure surface were evaluated through following formulae:

(9) $$d=\frac{\sigma_{C}\sigma_{b}\left(\sqrt{13}-2\right)}{\sigma_{C}-2\sigma_{b}}$$

(10) $$\beta =atan\left[\frac{3\left(\sigma_{C}-d\right)}{\sigma_{C}}\right]$$

where *σ_C_* and *σ_b_* are values assessed from crush test and Brazilian test, respectively, corresponding to failure force recorder at the test.

Once *d* and *β* were identified, cap surface parameters were evaluated by performing compression tests at several pressure levels and recording both axial and radial stresses. From these measurements, hydrostatic pressure *p* and equivalent von Mises stress *q* were computed, in order to identify the (*p*, *q*) point. The assessed point was on the cap surface, as yielding was active, providing one equation for the unknown quantities—cap eccentricity (*R*) and evolution pressure (*p_a_*). The second equation was obtained from the flow rule, assumed to be associative (i.e., plastic flow is perpendicular to the yield surface), usually adopted on the cap surface (see e.g., [14,26,33]), which yielded:

(11) R=(p−pa)2(1+α−αcosβ)23q

The outlined calculations led to the quantification of the listed four parameters corresponding to different relative densities. Here specifically, considered relative densities were: 0.73, 0.78, 0.83, 0.88 and 0.93. These values were further treated as “reference values” for verification purposes, regarding the estimates resulting from the proposed procedure.

## 5. Assessment of Modified DPC Model Parameters Through Proposed Inverse Analysis Procedure

**Figure 10:**
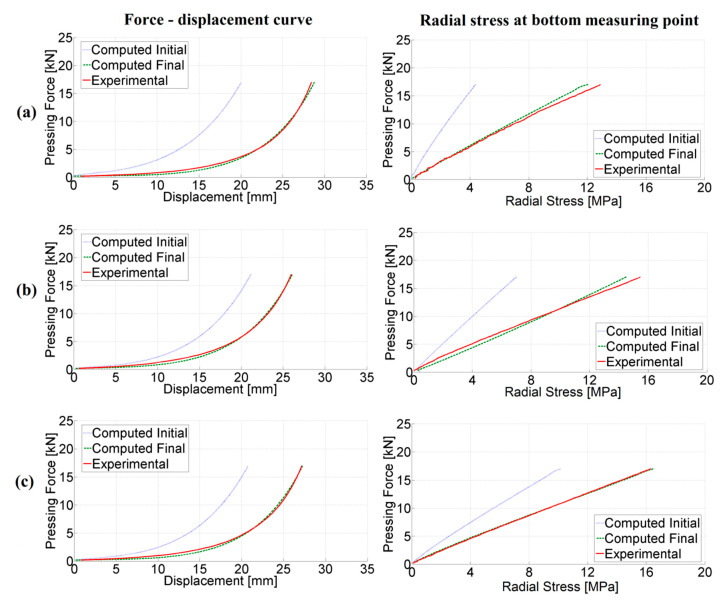
Comparison of experimental and computed measurable quantities from three tests: (**a**) configuration 1; (**b**) configuration 2; (**c**) configuration 3.

**Figure 11:**
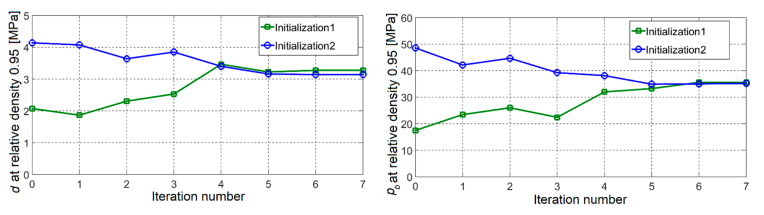
Convergence plots for two selected parameters.

**Figure 12:**
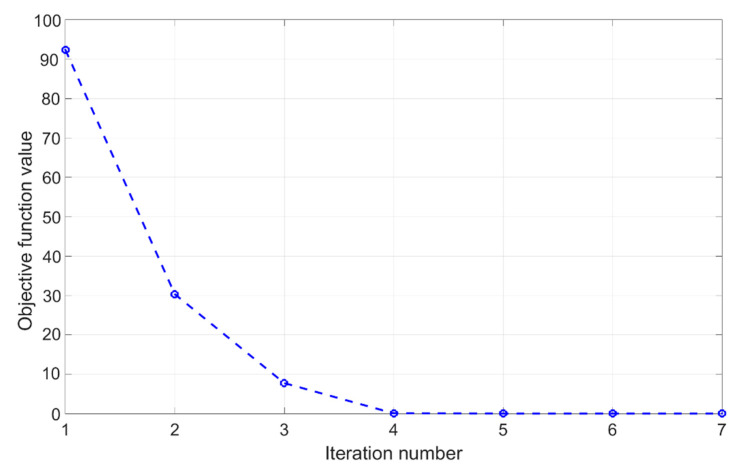
Convergence plots for the objective function.

The presented inverse analysis procedure was employed in order to assess parameters entering the modified DPC model using data collected from the compaction experiments by three different configurations (visualized in Figure 4) as inputs. From each experiment, the force–displacement curve and radial stress measurements in three points along the height are considered as measurable quantities. The formulated minimization problem turned out to be well-posed, and therefore efficiently solved by the trust region (TR) algorithm. A modified form of the “dog-leg” TR algorithm is employed here, with details of its numerical implementation given in [28]. The iterative minimization process involved up to 10 iterations in order to converge to the global minimum, evidenced by a good fit of experimental curves, as shown in Figure 10. Minimization was performed several times, starting from different initial values and converging all the time to the same parameter set. The convergence plots of the TR for the selected two parameters are visualized in Figure 11. Figure 12 shows the reduction of the objective function throughout iterations. The objective function is computed by employing Equation (3), where different quantities (i.e., the force–displacement curve and force–pressure curve) are accounted for through normalization. Other parameters (not shown here for brevity) evidenced similar convergence trends. The values of the parameters representing the solution of the inverse problem are listed in Table 3. For each parameter, the initial value, the final value and the exponent of the transition are given (see Equation (5)).

Convergence plots evidence that only seven iterations suffice to reach the global minimum of the objective functions. A similar result was also achieved with other initializations. This corroborates the conclusion of the proper selection of the minimization tool for the resulting inverse analysis. Here, the number of simulations involved in order to reach the result of the inverse analysis is approximately two orders of magnitude smaller than if, for example, artificial neural networks (ANN) were used, such as in [23]. As ANN involve a relatively large number of simulations for the phase of “training”, they, in general, involve a significantly larger number of simulations than mathematical programming algorithms. An alternative way of solving the resulting inverse problem could be by employing the adaptive collocation strategy within ANN, such as in [34]. To the best of our knowledge, such an approach is not yet applied within the context studied here.

**Figure 13:**
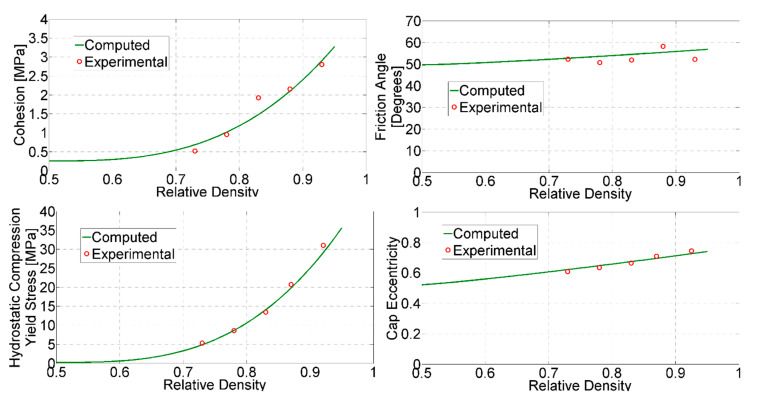
Comparison of assessed parameter values (continuous curve) against their values assessed through destructive tests (circles).

The selected parameters are compared against the reference values assessed through the procedure briefly outlined in Section 4. The comparisons are visualized in Figure 13. From the figure, it may be observed that for each considered parameter, the evolution with relative density is well captured. It is worth noting that the proposed parameter transition, given by (5), proves to be general enough to resemble both the exponential trend (observed for parameters *d*, *p_b_*, etc.) and the linear one (observed for parameter *β*).

**Figure 14:**
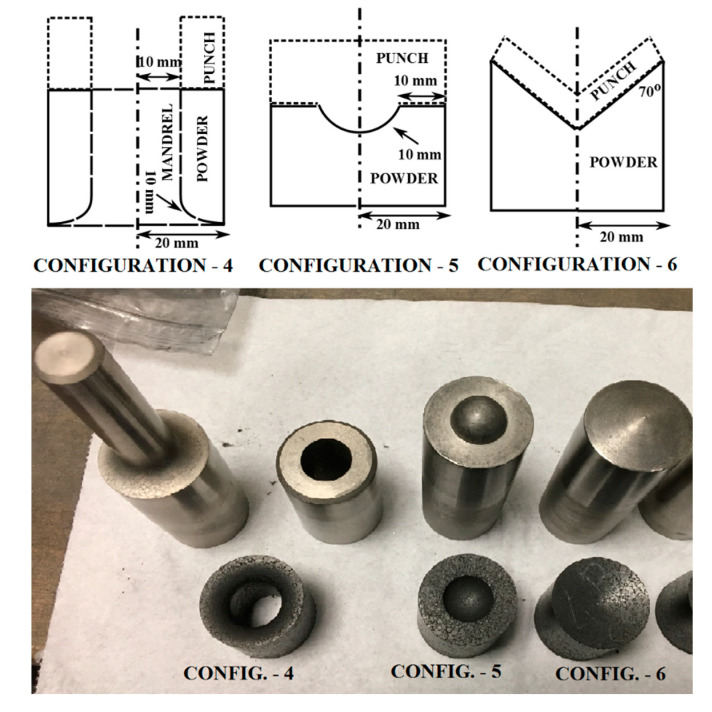
Die/punch configurations used for verification purposes.

**Figure 15:**
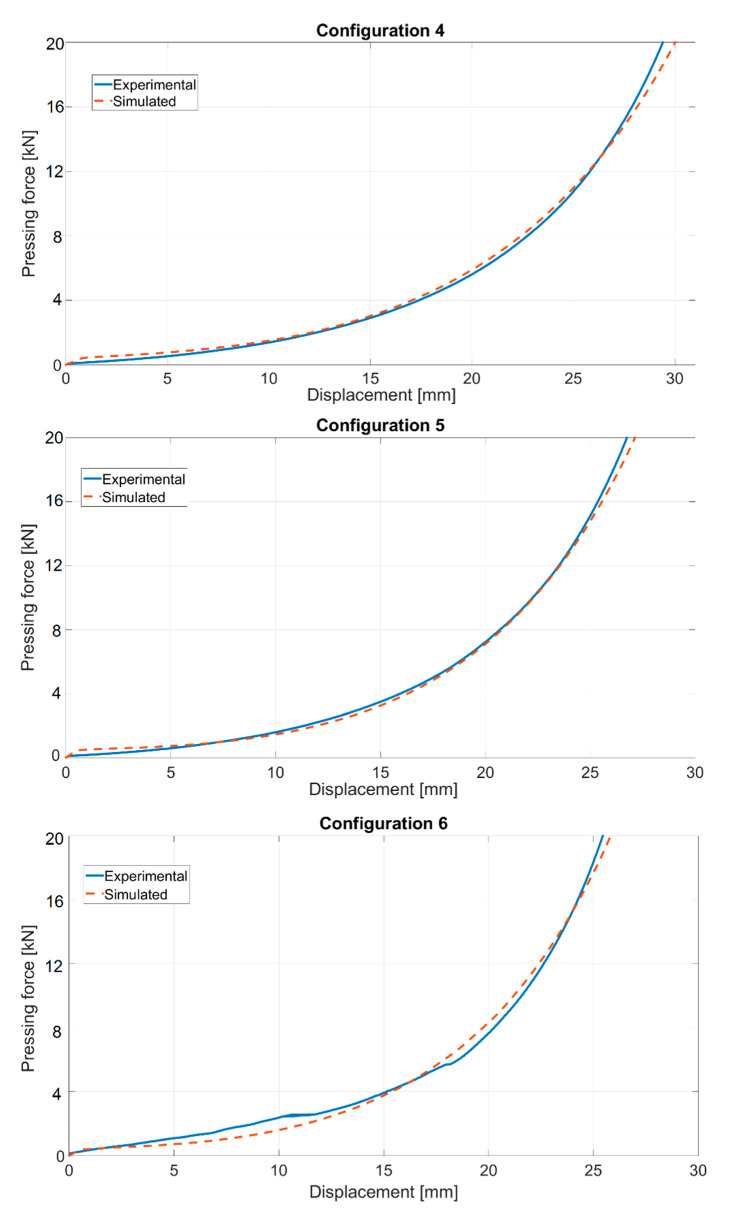
Force—displacement curves from three additional configurations (depicted in Figure 13).

Further comparative checks consisted of performing additional experiments and related numerical simulations, regarding the die/punch geometries that were different from those used within the calibration tests. To this purpose, three configurations were produced (see Figure 14) and employed within the compaction tests, which led to three additional force–displacement curves. The numerical simulations of all three compaction tests were performed attributing to the parameter values listed in Table 3. The comparisons of the experimental and numerical curves are visualized in Figure 15, showing extremely good agreement, corroborating the conclusion that the parameters assessed on the basis of the proposed inverse analysis procedure can indeed be treated as representative material parameters.

## 6. Conclusions

Complex constitutive models with a large number of governing parameters are frequently employed within the simulations of the ceramic powder compaction process. The calibration of such models through inverse analysis methodology proved to be rather promising, offering greater flexibility with respect to conventional testing methods. With the examples treated in this paper, it was demonstrated that parameters can be assessed directly as a function of relative density, exploiting a significantly smaller amount of experimental data compared to destructive tests performed on green bodies. Such an outcome may be advantageous from an industrial perspective, since it is more robust and economical, while, at the same time, it provides data for a wider range of relative densities. Within the examples treated in this paper, it was evidenced that the parameters are assessed considering a relative density starting from 0.5 up to 0.95. When assessing the same parameters through more laborious destructive tests, a larger number of experiments are required, with an additional limitation related to the difficulty in producing and testing green bodies with a low relative density (approximately 0.7 for the alumina powder tested here). Such a circumstance implies the need to perform an extrapolation of the assessed values outside of the treated range, potentially introducing additional errors to the estimates. With the proposed procedure based on an inverse analysis, such a limitation does not exist, while the parameters are assessed in a continuous manner over the complete range of relative densities considered in the compaction experiment.

Examples treated in this paper demonstrate that the proposed method offers a systematic framework for identifying representative material parameters, avoiding particular solutions that are capable of fitting only a single experiment. Such a result is ascertained by considering different geometries within the calibration phase, and, hence, stimulating large differences between stress states within the samples. The validity of the assessed properties is verified by comparing numerical and experimental curves from arbitrary shapes of die/punch configurations, which proved to be in excellent agreement.

Further developments currently in progress concern the comparisons of numerically predicted relative density distributions with the experimentally measured one. Such a quantity can be assessed through X-ray computed tomography measurements.

## Figures and Tables

**Table 1:** Selected parameter values assessed through inverse analysis procedure based only on force–displacement curve from compaction of cylindrical green body.

	Cohesion (*d*) *P*_0_ [MPa] *P*_1_ [MPa]		Hydrostatic Yield Stress (*p_b_*) *P*_0_ [MPa] *P*_1_ [MPa]	
Initialization 1	0.494	2.414	0.181	44.081
Initialization 2	0.435	3.087	0.149	25.933

**Table 2:** Sensitivity of measurable quantities to cohesion (*d*) at relative density 0.95.

Measurable Quantity	*q*_1_	*q*_2_	*q*_3_
Initialization 1	0.0040	0.0096	0.0234
Initialization 2	0.0668	0.0866	0.1551

**Table 3:** Parameters assessed through inverse analysis procedure.

Parameter	Initial Value	Final Value	Exponent *n*
Young’s modulus (*E*):	0.247 [GPa]	2.907 [GPa]	1.577
Poisson’s ratio (ν):	0.048	0.204	3.127
Cohesion (*d*):	0.257 [MPa]	3.274 [MPa]	2.909
Friction angle (*β*):	49.64 [°]	56.84 [°]	1.072
Cap eccentricity (*R*):	0.521	0.740	0.250
Hydrostatic yield stress (*p_b_*):	0.230 [MPa]	35.581 [MPa]	3.032
Coulomb friction coefficient:	0.082		

## Data Availability

Data sharing is not applicable for this article.
